# Modelling cultural responses to disease spread in Neolithic Trypillia mega-settlements

**DOI:** 10.1098/rsif.2024.0313

**Published:** 2024-10-16

**Authors:** R. Alexander Bentley, Simon Carrignon, Bisserka Gaydarska, John Chapman, Brian Buchanan, Michael J. O'Brien

**Affiliations:** ^1^ Department of Anthropology, University of Tennessee, Knoxville, TN 37996, USA; ^2^ Center for the Dynamics of Social Complexity, University of Tennessee, Knoxville, TN 37996, USA; ^3^ McDonald Institute for Archaeological Research, University of Cambridge, Cambridge CB2 3DZ, UK; ^4^ Department of Archaeology, Durham University, Durham DH1 3LE, UK; ^5^ Department of Geosciences, Eastern Washington University, Cheney, WA 99004, USA; ^6^ Department of History, Philosophy, and Geography and Department of Health and Behavioral Sciences, Texas A&M University–San Antonio, San Antonio, TX 78224, USA

**Keywords:** cultural evolution, epidemiology, ancient disease, susceptible-infected-recovered-susceptible, social distancing

## Abstract

As zoonotic diseases coevolved with early agriculture, social distancing within dense human settlements could have conferred a selective advantage in terms of infection risk. Here, we consider the case of Trypillia mega-settlements after 4000 BC, as virulent diseases began affecting humans in the Black Sea region. Through epidemiological susceptible-infected-recovered-susceptible (SIRS) models situated on clustered networks and on a site plan of a Trypillia mega-settlement, we show the adaptive benefits of decreasing either occupation density or the frequency of interactions with other communities across the settlement. We explore critical thresholds in these parameters that may shed light on the fluctuations of population densities at Trypillia mega-settlements before and after approximately 3600 BCE. Our findings suggest that disease was probably a significant driver of human settlement patterns by late Neolithic times.

## 1. Introduction

Infectious diseases are among the strongest selective pressures on human genetic evolution [1,2]. An ‘epidemiological transition’ probably began thousands of years ago, with early farmers living in close proximity to animals and their waste [3–5]. The earliest zoonotic pathogens include salmonella, measles, tuberculosis, viral hepatitis, cholera and typhoid [6–8]. The decimation that these diseases wrought for New World populations upon colonial contact [8–13] is indicative of the millennia of exposure that Eurasian populations had already experienced.

How did those Eurasian populations adapt to disease? Ancient DNA studies have indicated ‘no strong sweeps associated with immunological phenotypes’ over the last 8500 years [14] but also selection for genes related to metabolism, exposure to pathogens and inflammatory response [15–17]. As a form of gene–culture evolution [18,19], consuming dairy products might have helped lactose-tolerant individuals survive epidemics and famines [20].

In addition to genetic adaptations to Neolithic diseases, adaptive behaviours would have included avoidance of visible infection symptoms [21–23]. As a cultural norm, social distancing would have reduced disease-transmission rates. The trade-off is that too much social distancing, voluntary or otherwise, would negate the benefits of living closely together and/or congregating, including social support, collective knowledge and cooperative child rearing [24–27].

Social-distancing norms appear to post-date the strongly nucleated phases of early Neolithic villages, such as Çatalhöyük (*ca* 7100–5950 cal BC) in Anatolia, where several thousand people lived in a dense configuration of interconnected houses, with few signs of serious infectious disease [28]. Later, Çatalhöyük West (*ca* 6200 cal BC) was smaller and its houses more dispersed as the Neolithic spread into western Anatolia. By the sixth millennium cal BC, small, dispersed settlements predominated in Greece, the Southern Balkans and north and west of the Danube.

Zoonoses were evolving in the region at this time, including a typhoid-like progenitor of salmonella [29], tuberculosis [30–33] and plague-bearing *Yersinia pestis* [34]. Salmonella and bovine tuberculosis were probably transmitted via food rather than person-to-person contact [35].

Here, we explore the hypothesis that clustered Neolithic settlements were adaptive with respect to disease, in the process re-evaluating the evolutionary history of disease [36] as a significant driver of human adaptive behaviour. Specifically, the consequences of the appearance, from 4000 cal BC, of highly populated Trypillia ‘mega-settlements’, are uncertain. Inter- and intra-settlement clustering could have increased population resilience to zoonotic diseases [4,37], as containment of infections within clusters would have spared the larger population [38–41].

## 2. Diseases at Trypillia mega-settlements

As our case study, we consider a series of late Neolithic Trypillia mega-settlements (*ca* 4000–3400 cal BC) spread across approximately 250 000 km^2^ of the forest–steppe interfluve between the Bug and Dnieper rivers of west-central Ukraine. In the early Trypillia (pre-mega-settlement) phase (*ca* 4800−4300/4100 cal BC), settlements were less than 30 ha in size but later grew to as large as 320 ha [42–44]. Some of the largest settlements, such as Nebelivka (*ca* 3980–3780 cal BC), Taljanki (*ca* 3820–3610 cal BC) and Maidanetske (*ca* 3950–3630 cal BC) [45], were spaced 18–24 km apart, with each containing houses—almost 1500 at Nebelivka and perhaps as many as 3000 at Maidanetske [46]—arranged in concentric rings, with inner radial streets that led to a large open area. Each mega-settlement followed those ‘global’ planning principles, although there is considerable variation in how they were activated.

**Figure 1.**
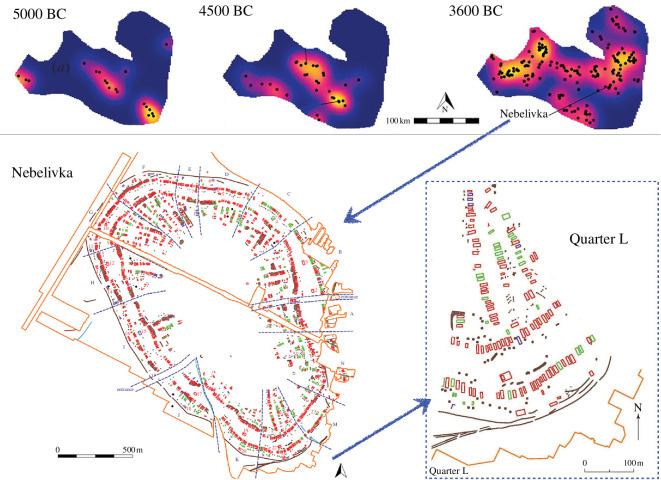
Multiple levels of Trypillia settlement clustering. Top, regional site distribution through time in quartiles [47]; colours range from blue (lowest density) to yellow (highest density), and black dots represent settlements. Bottom, interpretative plan of Nebelivka (*ca* 3980–3780 cal BC), zooming in on quarter L [45]; orange, limits of remote sensing; red, burnt structures; purple, unburnt structures; green, probable structures; blue dotted lines, quarter boundaries; assembly houses are numbered.

Mega-settlements comprised approximately a dozen pie-shaped quarters (segments) of 50–150 houses each (figure 1), typically with its own mega-structure, referred to as an assembly house [42,45–47]. Although there were global planning principles across Trypillia mega-settlements, quarters within the same settlement varied substantially in layout and use of space. At Nebelivka, 14 variations were identified, including the number of pits associated with a house, the number of houses in a neighbourhood and how they were situated relative to each other. Houses were made of heavy timber and had a typical size of 7.5 × 4 m, although there was considerable variation among quarters and neighbourhoods [48].

The presence of assembly houses in each quarter suggests they were the loci of population clustering through political organization [45,46]. This low-density clustering might help explain why, when looking at data on settlements in Romania, Moldova and Ukraine [49], the population density of Trypillia sites does not increase with site area, in contrast with most (but not all) cities, ancient to modern [50–53].

Estimated population densities of Trypillia mega-settlements through time are highly variable [46,54,55]. For the largest settlements, it is unclear whether populations—perhaps up to 10 000 people—inhabited them simultaneously or used them sequentially, with populations of a few thousand occupying only parts of a settlement at any one time [42–45,56–58]. By late in the fourth millennium BC, populations had declined and settlements became smaller, albeit with some still covering up to 60 ha [43,59].

Were these changes in settlement density the result of disease prevalence? Trypillia mega-settlements have been hypothesized to have been exposed to plague dispersion associated with Neolithic population decline in the fourth millennium BC [60]. Social distancing was a potential adaptation. Palaeopathological evidence from limited human remains recovered from Kosenivka suggests that by *ca* 3700 cal BC, ‘higher disease burden … may have prompted the shift to a dispersed settlement pattern with potentially better living conditions’ [59]. Dynamic response to disease risk might help explain the variation in estimated population densities of Trypillia mega-settlements through time.

Here, we model how food-borne diseases could have spread through these mega-settlements. The model is based on the proposal that assembly houses facilitated food sharing at the level of the quarter, which is a more conservative assumption but does not deny that finer-scale clustering at the level of neighbourhoods within each quarter was common. In fact, food-sharing networks may have been more clustered than were networks of general interaction, based on ethnographic studies of low-density sedentary societies [61,62].

## 3. Modelling disease spread at Nebelivka

As a way to think through the implications of a mega-settlement layout, consider the houses and the quarters within which they cluster at Nebelivka (figure 1). The probability of one household infecting another is essentially the probability of contact multiplied by the probability of infection upon contact. For example, imagine a high probability as 100% and a low probability as 10%. For food-borne illnesses such as salmonella—assuming food was shared primarily with immediate neighbours but not with those from other quarters—we might suppose the transmission rate to be close to 100% among four houses (high × high) versus only 1% between quarters (low × low). For airborne diseases, the infection rates among houses would also be a near certainty, but if the infection rate between quarters were of the order of 10% (low × high), the overall infection rate would be an order of magnitude higher than for food-borne diseases and would probably spread across the entire settlement population.

These considerations raise the hypothesis that Trypillia settlement layouts, as a result of the clustering of interaction within quarters, created a resistance to salmonella and food-borne tuberculosis. We can model potential thresholds between full recovery and endemic diseases at different levels of clustering. We start with a simple model of disease spread on a clustered network [38,63]. For parsimony, the parameters need only include the infectiousness of the pathogen as well as the network of social contacts and infection-recovery periods among agents [40,64]. Using parameters in table 1, each simulation generates an interaction network on which to run the susceptible-infected-recovered-susceptible (SIRS) model. The SIRS model is modified from [63], which we recoded in vector form to increase simulation speed in order to explore the parameter space. For models 1 and 2, each simulation generates a new interaction network, for the given number of quarters and households per quarter, which links households from the same quarter with probability p , and households from different quarters with probability q multiplied by a distance effect, $e^{-dx}$ . For model 3, the SIRS model is situated on a network generated on the spatial map of Nebevlika itself [45].

**Table 1.** Parameter values, building on those of [63], in the SIRS model (the asterisk * denotes 30 values logarithmically distributed in the range, and the dagger † denotes values derived from the archaeology).

parameter	model 1	model 2	model 3
quarters per settlement, $n_{q}$	10	{10,20}	14†
houses per quarter, $n_{h}$	{10,20,100}	{5,…,180} ${,}^{*}$	15–123†
total population size, N	{100,200,1000}	50–1800	{733, 690, 869}†
within-quarter contact rate, p	0.65	0.65	0.65
between-quarter contact rate, q	0.02 | {0.1,0.01,0.001}	{ $10^{-3},\ldots ,0.25$ } ${,}^{*}$	{ $10^{-3},\ldots ,0.25$ } ${,}^{*}$
distance effect, d , in $e^{-dx}$	0.3	0.3	0.3
infection rate, $S_{I}$	0.06	0.06	0.06
recovery rate, $I_{R}$	0.03	0.03	0.03
re-susceptibility rate, $R_{S}$	0.01	0.01	0.01

### 3.1. Model 1

**Figure 2.**
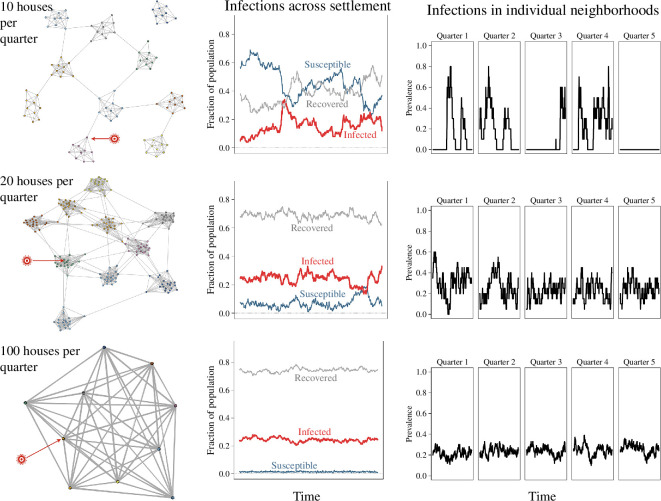
Aspects of the epidemic spread in three different clustered networks, representing 10 quarters of (top) 10 houses each, (middle) 20 houses each and (bottom) 100 houses each. The inter-quarter contact probability, q , was set to 0.02 and other variables are listed in table 1 under ‘model 1’. One infection (red) is introduced at one house to start the model. The epidemic across the whole settlement and the infections in five of the individual quarters.

At an abstract level, each node in the clustered network represents a household, and each cluster of nodes represents a quarter. Using the settlement map (figure 1) as a guide, we model 10 quarters as network clusters and test a range of quarter sizes from 10 to 100 houses in each (figure 2). To initiate each model run, one house becomes infected (red arrows in figure 2, *left*), and the infection spreads to another house within its quarter with probability p . Each household also has a smaller probability, q , of making a visit to another quarter (e.g. bringing contaminated food or fleas to another quarter’s assembly house). We consider a range of possible transmission rates, along with other essential parameters (table 1).

In this SIRS format, when a household recovers from infection, with probability $I_{R}$ , it is no longer a potential site of infection. Being recovered could represent the house being abandoned, self-isolating, burned down or full of recovered individuals with immunity. With probability, $R_{S},$ a recovered household can become susceptible again, which could represent a reoccupation of the house site or a willingness to risk exposure again. Each cluster is assigned a spatial coordinate, and the probability of contact between groups decreases with distance x via an exponential fall-off, $e^{-dx}$ , with fixed d (table 1).

Population density and social distancing are primary factors in the simulation results. Figure 2 shows the results when we assume 10 quarters with p=0.65 , q=0.02 and other parameters (table 1). Keeping p and q constant, figure 2 shows the effect of changing the number of occupied houses per quarter. With 10 houses per quarter (figure 2, *top*), there are outbreaks in multiple quarters, which generally recover from the infection. With 20 houses per quarter (figure 2, *middle*), the infection is still persistent but with notable ups and downs and different timelines between quarters. With 100 houses per quarter (figure 2, *bottom*), the infection is endemic, with approximately a sixth of the whole settlement and of each quarter infected through time.

**Figure 3.**
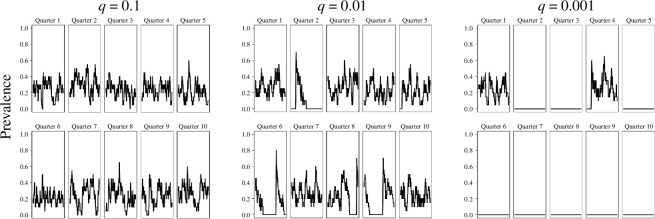
The effect of varying the rate of inter-quarter contact, q , while keeping p constant and the population density at 20 houses per quarter. The timelines for 10 quarters are shown in each of the three panels.

Next, we illustrate the effect of varying the rate of inter-quarter contact, q , while keeping p constant and the population density at 100 houses per quarter (figure 3). For example, with 20 houses per quarter, the disease persists in all 10 quarters with q=0.1 and q=0.01 , but with q=0.001 , the disease is contained within a couple of quarters and the others remain uninfected. This containment is achieved by reducing the rate of interaction between quarters by an order of magnitude.

### 3.2. Model 2

Having illustrated how reducing population density and/or strict norms of social distancing could have mitigated disease spread in Trypillia settlements, the next step is to explore the dynamics more generally, with hundreds of simulations at different parameter combinations. The key parameters we test are the density of houses per quarter and the probability of significant interaction with another quarter. Other parameters are kept constant (table 1).

**Figure 4.**
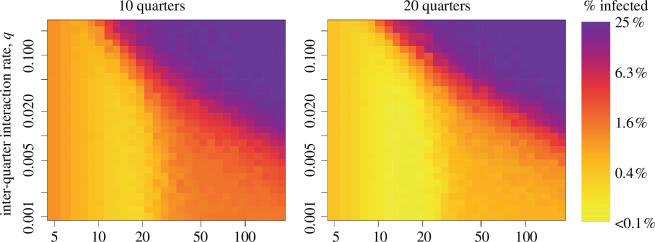
Simulation results of the SIRS model for each of 900 (30 × 30) different parameter combinations, averaging 200 runs at each parameter combination, for a total of 180 000 simulations per panel. On the vertical axis is the chance of making contact with another quarter ( q ), versus settlement density (occupied houses per quarter) on the horizontal axis. Colours show the percentage of the population that is still infected at the end of the simulation, with lighter yellow indicating almost no infected people, orange indicating one community infected and violet indicating an outbreak across the entire population.

Representing the mean over 200 simulations at each of 900 different parameter combinations, figure 4, *left* shows that as the likelihood of contacting another quarter increases, there is a relatively sharp transition between endemic infection across the mega-settlement (purple) and a scenario where the disease disappears (yellow) or has affected only a limited number of houses within a few quarters (orange). The clustering accommodates growth in the overall population, in the sense that when we double the number of quarters (clusters), the respective zones of risk shift towards higher densities of houses (figure 4, *right*). The *y*-axis in each plot of figure 4 confirms social distancing from other quarters.

### 3.3. Model 3

**Figure 5.**
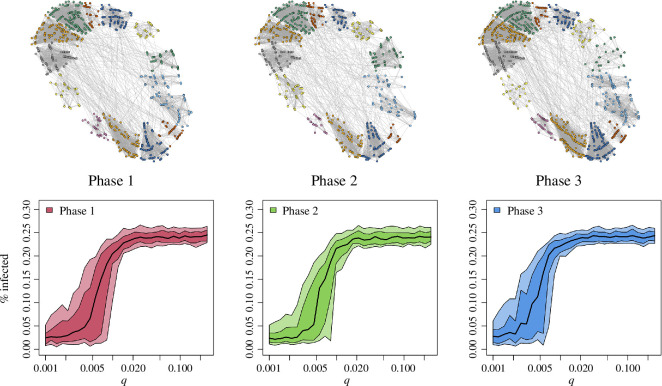
Infections modelled on the site of Nebelivka, for the three chronological phases as shown in figure 1. The dots represent actual households on the site plan (coloured by quarter), and links represent the simulated interactions under one example run of a SIRS model (each run has a unique network of interactions). Also shown are the results of each of the 30 values of the inter-quarter interaction rate, q , versus the per cent infected at the end of the simulation: the black curve shows the mean of 200 simulations and the coloured bands show 75% and 50% density intervals.

Finally, we situate the SIRS model on a GIS map of Nebelivka (figure 5), using the quarter assignments as determined previously through archaeological fieldwork [45]. After we ran 200 simulations at 30 different values of inter-quarter interaction rate, q , figure 5 shows the change in the fraction of infected households at the end of each simulation (mean of 200 simulations and confidence intervals). The results for the three chronological phases are similar, each with a relatively abrupt transition in proportion infected at approximately q=0.01 . The transition is particularly sharp in the lower end of the range of results, that is, as q is increased above 1%, there suddenly are almost no simulations that finish without infected households. Decreasing q below 1%, it quickly becomes quite possible to finish without infected households, as the worst-case scenarios reduce in magnitude as well. Note the results in figure 5 show the fraction infected at the last time step of the simulation, and because endemic infection courses through waves (figure 2), the maximum infection rates shown (above 25%) represent the majority of the population experiencing infection.

## 4. Discussion

Our simulations confirm that although higher population density facilitates disease spread between quarters, this can be counteracted by social distancing to reduce visits between quarters. Modular growth of mega-settlements, yielding discrete clusters around assembly houses, is consistent with low-density urban patterns. If social distancing occurred at the group level, where one group was attuned to the disease being present in another group, our simulations show how a disease at a Trypillia mega-settlement could persist in one quarter but not in another. This was expected, but surprising were the thresholds in the parameters that triggered abrupt changes in disease spread versus containment. This was evident when we modelled Nebelivka, which suggests the clustered Trypillia settlement pattern was adaptive against the spread of diseases such as salmonella. It is quite possible that food-sharing networks at Trypillia settlements were clustered at the neighbourhood scale—each quarter comprises multiple neighbourhoods [65]—which would have meant even more protection against community-wide epidemics than we have tested here.

As population density increased, other adaptive practices could have included domestic hygiene, dairy consumption, deliberate house burning and waste-disposal practices. The proximity of dairy cattle, sheep, goats and pigs, together with the quantity of dung they produced [66], could have been highly problematic to the inhabitants. In addition, the risk of pests inside houses would have increased significantly with food storage (e.g. cereals and pulses) and cooking [65]. This web of interrelated disease vectors would have necessitated ‘an effective refuse management strategy—one that quickly removed household food discard and, consequently, disease-carrying vermin from the settlement’ [46]. At Trypillia mega-settlements, the deliberate burning of timber houses was a regular practice [67]. At Nebelivka and Maidanetske, approximately two-thirds of the houses—over 1000 and 2000 houses, respectively—were deliberately burned [46,65]. Regardless of whether or not there was a ritual significance to the destruction, regular burning would have served to prevent disease by eliminating pests [67].

Such cultural practices were inherited over long periods of time [68]. Thousands of years before Trypillia, at Çatalhöyük (*ca* 7100–5950 cal BC), floors were regularly swept and replastered, walls were repainted, and there was careful burial of the dead [69]. Subsequent adaptations in settlement spacing would have lasting effects on the demographic and social development of Neolithic societies. The peak of settlement nucleation in the Balkans and the Hungarian Plain in the early to mid-fifth millennium cal BC was followed by the dispersal of homestead-size settlements in the late fifth to fourth millennia cal BC, in contrast with the Trypillia group increases in settlement size up to 320 ha.

Subsequent population dispersal into Europe in the Chalcolithic to early Bronze Age involved even lower population densities. Later, at Maidanetske, $\delta^{15}$ N patterns in cattle bones [46] suggest a possible shift to transhumant pastoralism. Notably, the rise of mobile pastoralists in the region featured prominently in the dispersal events and cultural-technological change in the late fourth millennium BC [70].

Whether these developments were adaptations to disease is an open question. Zoonoses stemming from food produced from domestic herds can spread within herds interacting in the wider orbit of the settlement [71]. Plague-bearing *Yersinia pestis* strains that date *ca* 5000–3000 cal BC in the Cis-Baikal region of Siberia and elsewhere in northeast Asia [8,34,60,72–74] have been hypothesized as contributing to a high disease burden that led populations into more-dispersed settlements [59].

No matter the role(s) the mega-settlements played in the greater Trypillia socio-cultural sphere, or the precise reason(s) for the tremendous growth in settlement size after 4000 cal BC, interaction networks spanned the occupation of the forest–steppe of Ukraine well before then [47]. We propose that the individuals who inhabited the larger settlements, whether year-round or seasonally [47], needed to maintain socio-economic network ties while also minimizing infection risk. At a mega-settlement, the assembly houses facilitated socio-economic ties via large social gatherings at the cost of risking the spread of disease. The benefits included food sharing—crucial for households whose crops had failed or who had lost grazing rights to a piece of land—but the risk lay in food-borne diseases. In terms of settlement density and interaction between quarters, this minimax problem—how to minimize the maximum loss—might be optimized in the boundary zone of figure 4.

## 5. Conclusion

New evidence for infectious diseases and their etiological agents in Neolithic and Bronze Age contexts has raised new hypotheses regarding the effects of prehistoric cultural and behavioural responses to pathogen presence, diversity and evolution. In these early settlements, not only was there a reliance on wide-ranging exchange networks, which could have increased the risk of infection significantly, but clustered communities would have faced a trade-off between reducing disease-transmission rates while maintaining the social benefits of living closely together. One proposed response is social distancing within dense settlements, which would have conferred a selective advantage in terms of lowering exposure to disease and might help explain why low-density urbanism characterized the world’s first ‘cities’.

To examine this hypothesis, we focused on Nebelivka, one of many Trypillia mega-settlements located in the forest–steppe region of Ukraine occupied *ca* 4000–3400 cal BC—a date range that encompassed the period when virulent diseases began affecting populations in the Black Sea region. We used epidemiological (SIRS) models that were built around clustered networks and a site plan of Nebelivka to examine the epidemiological benefits of decreasing either the population density or the frequency of interactions among segments of the settlement. We identified critical thresholds in the clustering of houses within neighbourhoods, and of neighbourhoods within quarters, mitigated against epidemics. This suggests settlement clustering was an adaptive behaviour during the Neolithic, sparing populations from widespread zoonotic diseases.

## Data Availability

The data and code used for the analyses are available at [75].
